# Physical Activity and Sociodemographic Profile of Brazilian People during COVID-19 Outbreak: An Online and Cross-Sectional Survey

**DOI:** 10.3390/ijerph17217964

**Published:** 2020-10-29

**Authors:** Rodrigo L. Vancini, Luiz Camargo-Neto, Claudio A. B. de Lira, Marília S. Andrade, Ricardo B. Viana, Pantelis T. Nikolaidis, Beat Knechtle, Paulo Gentil, Luiz H. V. Piacezzi, Maria C. B. Teixeira Lopes, Ruth E. Assayag Batista, Cássia R. Vancini-Campanharo

**Affiliations:** 1 Centro de Educação Física e Desportos (CEFD), Universidade Federal do Espírito Santo (UFES), Espírito Santo (ES) 29075-910, Brazil; rodrigoluizvancini@gmail.com; 2 Escola Paulista de Enfermagem (EPE), Universidade Federal de São Paulo (UNIFESP), São Paulo (SP) 04023-062, Brazil; camargo.luiz@unifesp.br (L.C.-N.); piacezzi@unifesp.br (L.H.V.P.); lopes.carolina@unifesp.br (M.C.B.T.L.); ruth.ester@unifesp.br (R.E.A.B.); vcassia@hotmail.com (C.R.V.-C.); 3 Setor de Fisiologia Humana e do Exercício, Faculdade de Educação Física e Dança, Universidade Federal de Goiás (UFG), Goiânia, Goiás (GO) 74.690-900, Brazil; andre.claudio@gmail.com (C.A.B.d.L.); vianaricardoborges@hotmail.com (R.B.V.); paulogentil@hotmail.com (P.G.); 4 Departamento de Fisiologia, Universidade Federal de São Paulo (UNIFESP), São Paulo (SP) 04023-910, Brazil; marilia1707@gmail.com; 5 School of Health and Caring Sciences, University of West Attica, Athens, 11521 Attica, Greece; 6 Medbase St. Gallen Am Vadianplatz, St. Gallen and Institute of Primary Care, University of Zurich, 8091 Zurich, Switzerland; beat.knechtle@hispeed.ch; 7 Liga de Hipertensão Arterial, Universidade Federal de Goiás (UFG), Goiânia, Goiás (GO) 74.690-900, Brazil

**Keywords:** physical activity, COVID-19, pandemic, sociodemographic, online survey

## Abstract

The Coronavirus Disease 2019 (COVID-19) outbreak has created an unprecedented impact on global health and further aggravated the physical inactivity pandemic. For this reason, the understanding of sociodemographic variables in the context of physical activity levels are important for the field of public health in order to assist in relevant public health decisions. Our main aim was to characterize sociodemographic variables and physical activity levels and their association with COVID-19 aspects. We applied an online Google survey with closed questions in Brazilian people of different age and regions, both sexes and physical activity levels (*n* = 1.726). Our main results were that participants who had symptoms of COVID-19 had the highest percentage of level 1 of physical activity (the lowest level according to the classification used) and those who showed no symptoms had the highest percentage of levels 2 and 3 of physical activity; that is, close to the light/moderate levels of physical activity. This cross-sectional study in the Brazilian population provided important sociodemographic data and COVID-19 aspects regarding the level of physical activity. It is possible to assume that the regular practice of physical activity could positively impact health status and quality of life and be a tool in the field of public health to cope (from a physical and mental point of view) with disease scenarios that require quarantine.

## 1. Introduction

The Coronavirus Disease 2019 (COVID-19) pandemic has had an unprecedented impact on global health, economy and functioning of societies [1]. Currently (25 September 2020), there are 32,110,656 million confirmed cases of coronavirus in the world, with 15,987,906 million cases in the Americas and 4,624,885 million in Brazil [2]. However, this scenario is dynamic and changes every day. The highest number of cases have been observed in the United States of America, India, Brazil, the Russian Federation, Peru and Colombia [2]. 

In Latin America countries, especially in Brazil (with 138,977 confirmed deaths on 25 September 2020), which is an emerging country with a high level of social inequality, and in urban and densely populated areas, the health and economic effects will be even more harmful and deleterious [2,3]. 

COVID-19 is an infectious disease caused by the severe acute respiratory syndrome coronavirus 2 (SARS-CoV-2), which mainly affects the pulmonary and cardiovascular systems [4]. Factors linked with an increased risk of mortality in COVID-19 include comorbidities such as obesity, diabetes, cardiovascular disorders and a lower functional capacity [5,6,7,8]. Poor lifestyles such as physical inactivity could worsen this scenario as it is related to impaired immune function and the increased risk of illness [9,10,11]. 

Conversely, physical activity could be an important tool in the prevention and treatment of non-communicable diseases associated with increased COVID-19 mortality [10,11]. Moreover, moderate levels of physical activity could improve the health status and quality of life and prognosis of patients infected with COVID-19 because it meliorates immunity and health-related physical fitness [5,12]. Nieman et al. [8] showed that higher levels of physical fitness reduced days with an upper respiratory tract infection and the severity of symptoms during the winter and fall, which are common cold seasons.

Maintaining an active lifestyle during social isolation is very important for general health but especially for those with risk factors and morbidities such as elderly people [6,13,14,15]. Physical activity might be essential because the elderly is the main risk group and needs to maintain social isolation. In order to maintain the levels of physical fitness and good physiological functioning to preserve most organic systems including the immune system and to face the mental and physical consequences of COVID-19 [6,16], regular physical activity practice is essential. 

The COVID-19 pandemic brought up the discussion of the importance of having healthy habits because social isolation could have a negative impact on physical activity levels, quality of life and health-related physical fitness [17,18]. In this regard, an international online survey found that the sitting time increased in the range of 5–8 h/day as a result of the COVID-19 outbreak [19]. Goethals et al. [20] showed the number of seniors attending physical activity programs during the COVID-19 outbreak decreased significantly and concluded that public health campaigns are necessary to communicate how important it is for older adults to maintain adequate physical activity levels at home. Constandt et al. [21] showed that self-reported physical activity levels decreased during the COVID-19 lockdown among Belgian people. This was associated with having less time available, sitting more and missing a familiar environment. A demographic cross-sectional study in Portuguese adults showed that strategies for promoting well-being during periods of social isolation should consider lifestyle habits according to the gender or age [22]. It seems important to understand the impact of these changes in the population in order to support public health policies and decisions.

Additionally, understanding the profile of sociodemographic variables is important for health and public authorities to make decisions to minimize the effects of the COVID-19 pandemic [23]. The assessment of sociodemographic variables (such as sex, age, income and education level) will allow the analysis of the negative impact of social differences and inequality on the effects of the COVID-19 pandemic [24] mainly in a country with serious social inequalities such as Brazil. 

For these reasons, the aim of the present study was to present the sociodemographic profile (age, sex, state and city of residence, marital status, religion, income and education level) of a convenience sample of Brazilian people according to physical activity levels and the relationship with COVID-19 aspects. Our hypothesis is that lower levels of physical activity place people at greater risk for presenting symptoms and infection by COVID-19.

## 2. Materials and Methods 

This study was an online survey designed and applied via Google Forms. Data collection was carried out in May 2020 from day 1 to day 31. It was at the moment when the scenario in Brazil was with 374,898 confirmed cases and 23,473 deaths and under rigid social isolations measures such as the closure of schools, gyms and places that could induce agglomeration as well as restrictions for public gathering, social distancing and the mandatory use of face masks [2,25,26]. When this article was written (September 2020), Brazil ranked second in the world in terms of the number of deaths due to COVID-19, with the USA in the first place [2,27]. 

Our convenience sample consisted of adults (women and men) from different regions of Brazil (*n* = 1726 respondents). The average age of our total sample was 40.5 ± 11.2 years, the mean of the group <60 years (37.2 ± 8.7, *n* = 1521) and ≥60 years (65.7 ± 3.9, *n* = 205). The inclusion criteria were to be Brazilian, over 18 years old and to accept the study terms:

“You are being invited to participate, as a volunteer, in the study “Evaluation of the knowledge of the Brazilian population about COVID-19 through an online survey”. We believe it is important to know about the population’s knowledge in order to develop preventive measures and try to contain the spread of the virus”.

The exclusion criteria were not answering all of the research questions and not completing the questionnaire according to the proposed aims. An online questionnaire (https://forms.gle/vzWqCCJ5dD4UYFVDA) was created with closed questions about the participant’s individual and sociodemographic characteristics, which were (1) age, (2) sex, (3) state and (4) city of residence, (5) marital status, (6) religion, (7) income, (8) education level and (9) physical activity levels. Multiple choice questions about COVID-19 were also included, which were (10) presence of symptoms of COVID-19, (11) diagnosis of COVID-19 and (12) health professionals (yes or no); if yes, (13) worked with people affected by COVID-19. Our questionnaire was created from the verification of issues listed from scientific literature and practical experience mainly in the field of epidemiology and public health associated with the pandemic of COVID-19.

The physical activity level profile during the COVID-19 pandemic was evaluated according to the following question: “On a scale of 1 to 5, being 1 (I am very little, not very physically active) and 5 (I am very, very physically active) that would be given today—in this exact moment—regarding your level of physical activity?” The questionnaire (with 13 questions) was prepared and made available on the online platform Google Forms and disseminated through social networks in order to reach the largest number of people possible in a short period of time. Our questionnaire was published on Facebook and Instagram and was shared in personal and professional profiles.

All participants read and agreed with the research procedures. The research was evaluated and approved by the university’s ethics committee and followed Helsinki’s principles for human studies (CAAE–31540620.9.0000.5505).

### Statistical Analysis

For the descriptive analysis of categorical variables, frequency (*n*) and percentage (%) were calculated. To compare the level of physical activity with the variables of interest, the chi-squared test was used. When necessary, the Likelihood Ratio test was used. A significance level of 5% (*p*-value < 0.05) was used. The data were analyzed using the Statistical Package for the Social Sciences (SPSS), version 19 (Chicago, IL, USA). 

## 3. Results

Table 1 shows sociodemographic data (present by absolute (*n*) and relative (%) values) categorized by physical activity levels. More than one third of participants referred to level 1 (lowest classification according to the classification criteria used) of physical activity and only 6% referred the highest classification (level 5). 

The same pattern was observed with regard to questions, the answer (No) being the most prevalent: Have you experienced symptoms of COVID-19? 84.5%; Did you have a confirmed diagnosis for COVID-19? 98.2%; Are you a health professional? 64.4% but “yes” was also expressive (35.6%); and If so, are you assisting people with COVID-19? 67.9% but “yes” was also expressive (32.1%). 

Table 2 shows the comparison of sociodemographic variables regarding COVID-19 in relation to the level of physical activity. The results in bold presented the highest absolute (*n*) and relative (%) values in the different categories and possibilities of answer. It was observed that participants who showed symptoms of COVID-19 had a higher percentage of level 1 (36.6%) of physical activity when compared with participants who did not show symptoms of COVID-19. Participants who did not show symptoms of COVID-19 had a higher percentage of levels 2 (21.1%) and 3 (25.8%) of physical activity than participants who showed symptoms of COVID-19.

## 4. Discussion

The aim of the present study was to present the sociodemographic profile of Brazilian people according to the current level of physical activity (*n* = 1726) during the COVID-19 outbreak. Our main results were that the majority of participants who showed symptoms of COVID-19 were those who presented level 1 of physical activity (36.6%), which, according to our classification criteria, was the lowest level of physical activity. Most participants who did not report symptoms of COVID-19 were those who reported a higher percentage of level 2 (21.1%) and 3 (25.8%) of physical activity; that is, from low to moderate level. This preliminary result, despite having to be better understood, may point out that the maintenance of some level of physical activity is associated with a lower presence of symptoms of COVID-19.

One possible explanation for the association of COVID-19 symptoms and physical activity level is that the regular practice of physical activity, as well as high levels of physical capacity, are related to an improved immune function and a decrease in immunosuppression and chronic inflammation associated with the aging process and subsequently lower risk of illness and pulmonary infections [8,28,29]. The lack of protection for COVID-19 symptoms in those with higher levels of physical activity might be explained by the J shaped curve that reflects the association between physical activity and immune function and risk of illness, with increased risk for both those who have very low and very high levels of exercise [7]. 

However, we should highlight that our study was not able to establish a causal relation; therefore, it might also be possible that people with COVID-19 symptoms were less prone to perform physical activity due to functional limitations or fear. 

Notwithstanding, our results suggest that regular physical activity might be recommended by health professionals and health authorities. In this regard, previous studies presented suggestions of how to perform physical activity during the COVID-19 pandemic with special attention paid to resistance training [15], exergames [16] and interval training [14]. These protocols usually involved home based activities and also highlighted their use on special populations [13,30], which allows the respecting of the measures of isolation and social distance.

Dowd et al. [23] pointed out that understanding the profile of sociodemographic variables is important for governments to make policy decisions rapidly and mitigate the negative effects of the COVID-19 pandemic. With the course of the COVID-19 pandemic, through sociodemographic studies it was possible to verify that the most serious cases and deaths were concentrated in people aged 60 years or older as well as with morbidities. It should be noted that 11.9% (*n* = 205) of our sample was people aged 60 or over and with low levels of physical activity. This is very important information for health systems around the world. 

This information becomes even more interesting because physical inactivity is a pandemic by itself and is the fourth leading cause of death worldwide [10,31]; a problem that was probably accentuated by the COVID-19 pandemic [32]. Hall et al. [32] pointed out that the world is confronted with two pandemics at the same time: COVID-19 and physical inactivity. In this sense, aggressive efforts need to be taken to get people physically moving during and after COVID-19 [13,14,15,16,32]. After all this chaos it is important that humanity learns lessons and improves health outcomes under normal conditions and resiliency and material and human infrastructure in the context of health and education to future pandemics.

A fact that was clear in our sample was that for almost all sociodemographic variables and the questions associated with COVID-19, the answers focused more on level 1 of physical activity, which was the lowest level of classification in our sample. We observed, in a statistically significant way and in a large sample (*n* = 1726), that participants who presented symptoms of COVID-19 were also those who reported a higher percentage of level 1 of physical activity (36.6%) and those who did not present were those who reported levels 2 (21.1%) and 3 (25.8%) of physical activity. This information per se can help public managers in the construction of public health policies that reinforce the importance of moderate levels of physical activity for a healthier society. Currently, there is nothing more powerful than the acquisition and processing of information, including sociodemographic data, to assist in coping with situations such as this. As Subramanian and James [33] pointed out, using an existing rich data-science infrastructure, countries can provide vital data and insights to guide appropriate health actions to COVID-19 and to prepare for the next pandemic. 

### Study Limitations and Practical Applications

As in all questionnaire (and cross-sectional) studies, the answers involve subjective and personal perceptions and are only a “snapshot of reality”. In a scenario such as this of collective and individual social insecurity it is possible that perception is altered. We also did not use a validated questionnaire to assess the level of physical activity. We used only one question in order to classify levels 1 to 5 (i.e., from very, very little to very, very high). Our intention in doing so was to increase adherence and facilitate people’s understanding because, in general, questionnaires to assess the level of physical activity are long and often require the assistance of trained professionals. Despite being an important limitation, it is not a fatal concern because we applied the questionnaire on a large scale. With this study, a large sample was able to understand and provide important sociodemographic data related to some aspects of COVID-19 in relation to the levels of physical activity despite the possible regional differences in Brazil. This could help to cope with the current scenario as well as in future pandemics. Other important limitations are the impossibility of establishing causal relations between variables and the limitation of reaching only people that have access to internet and are familiar with online questionnaires.

In addition, with the assessment of sociodemographic variables it is possible to see how much social differences and inequality influence the scenarios such as those provided by the COVID-19 pandemic. In the absence of adequate social protection and welfare programs aimed at people in a state of social vulnerability during the pandemic, countries in situations such as Brazil need to implement flexible and effective policies and legislation in order to minimize any harmful effects. These impacts may vary according to sex, age, income and education level as well as access to public and private health services [24]. This whole scenario has a significant impact on the state of health and quality of life individually and collectively. 

Our next and prospective steps are to understand the impact of age, socioeconomic status and social inequality, which is unfortunately very serious in Brazil and has a significant impact on the mortality and morbidity of patients who have been infected by the disease and living in different regions, in terms of the level of physical activity and the effects of social isolation on mental health and level of physical activity and different exercise types [34] in health, risk groups, and people aged 60 or over.

## 5. Conclusions

In our view, to improve health outcomes regarding the multiple negative effects of the COVID-19 pandemic, public managers should be guided by scientific data for political decisions. This should also extend to the post-pandemic of COVID-19. It is likely that the pandemic of physical inactivity has worsened even more and that social isolation will have harmful effects on the mental and physical health of the Brazilian population. In addition, Brazil’s brutal social inequality only further aggravated this scenario. Our main result was that people who had symptoms of COVID-19 were those who also had the highest prevalence of level 1 of physical activity (lowest) and those who did not show symptoms were those who had the highest percentage of levels 2 and 3 of physical activity. Thus, it is possible to assume that higher levels of physical activity could positively impact individual and collective health and be a tool in the field of public health to cope with scenarios such as the one we are experiencing. This cross-sectional study in the Brazilian population provided important sociodemographic data and COVID-19 aspects regarding the level of physical activity, which could help in facing the current and future similar scenarios. 

## Figures and Tables

**Table 1:** Profile of sociodemographic variables in relation to the participants’ physical activity levels.

Variables	Physical Activity Levels					Total
	1 (*n* = 620)	2 (*n* = 391)	3 (*n* = 425)	4 (*n* = 187)	5 (*n* = 103)	*n* = 1726
	35.9%	22.7%	24.6%	10.8%	6%	100%
Age group (years)						
<60 (37.2 ± 8.7, *n* = 1521)	583 (94.0%)	355 (90.8%)	344 (80.9%)	151 (80.7%)	88 (85.4%)	1521 (88.1%)
≥60 (65.7 ± 3.9, *n* = 205)	37 (6.0%)	36 (9.2%)	81 (19.1%)	36 (19.3%)	15 (14.6%)	205 (11.9%)
Sex						
Women	482 (77.7%)	301 (77%)	323 (76.0%)	132 (70.6%)	74 (71.8%)	1312 (76.0%)
Men	138 (22.3%)	90 (23.0%)	102 (24.0%)	55 (29.4%)	29 (28.2%)	414 (24.0%)
Brazil region						
North	6 (1.0%)	8 (2.0%)	5 (1.2%)	0 (0.0%)	3 (2.9%)	22 (1.3%)
Northeast	24 (3.9%)	10 (2.6%)	15 (3.5%)	10 (5.3%)	5 (4.9%)	64 (3.7%)
Midwest	19 (3.1%)	11 (2.8%)	16 (3.8%)	11 (5.9%)	6 (5.8%)	63 (3.7%)
Southeast (where São Paulo is located)	537 (86.6%)	341 (87.2%)	367 (86.4%)	158 (84.5%)	82 (79.6%)	1485 (86.0%)
South	34 (5.5%)	21 (5.4%)	22 (5.2%)	8 (4.3%)	7 (6.8%)	92 (5.3%)
Marital status						
Married	326 (52.6%)	202 (51.7%)	215 (50.6%)	91 (48.7%)	49 (47.6%)	883 (51.2%)
Divorced	42 (6.8%)	25 (6.4%)	35 (8.2%)	15 (8.0%)	9 (8.7%)	126 (7.3%)
Single	196 (31.6%)	117 (29.9%)	119 (28.0%)	58 (31.0%)	32 (31.1%)	522 (30.2%)
Stable union	49 (7.9%)	45 (11.5%)	42 (9.9%)	17 (9.1%)	9 (8.7%)	162 (9.4%)
Widowed	7 (1.1%)	2 (0.5%)	14 (3.3%)	6 (3.2%)	4 (3.9%)	33 (1.9%)
Religion						
Catholic	278 (44.8%)	169 (43.2%)	214 (50.4%)	99 (52.9%)	58 (56.3%)	818 (47.4%)
Evangelical	101 (16.3%)	60 (15.3%)	70 (16.5%)	24 (12.8%)	20 (19.4%)	275 (15.9%)
Spiritism	108 (17.4%)	64 (16.4%)	61 (14.4%)	24 (12.8%)	9 (8.7%)	266 (15.4%)
Agnostic and Atheist	70 (11.3%)	50 (12.8%)	34 (8%)	21 (11.2%)	9 (8.7%)	184 (10.7%)
Others	63 (10.2%)	48 (12.3%)	46 (10.8%)	19 (10.2%)	7 (6.8%)	183 (10.6%)
Total family income in minimum wage (BRL)						
Less than 1	24 (3.9%)	13 (3.3%)	16 (3.8%)	5 (2.7%)	5 (4.9%)	63 (3.7%)
1 to 3	170 (27.4%)	93 (23.8%)	105 (24.7%)	37 (19.8%)	20 (19.4%)	425 (24.6%)
4 to 7	180 (29.0%)	125 (32.0%)	124 (29.2%)	43 (23.0%)	34 (33.0%)	506 (29.3%)
8 to 10	88 (14.2%)	48 (12.3%)	68 (16.0%)	27 (14.4%)	12 (11.7%)	243 (14.1%)
More than 10	158 (25.5%)	112 (28.6%)	112 (26.4%)	75 (40.1%)	32 (31.1%)	489 (28.3%)
Education level according to the Brazilian standard						
Incomplete elementary school	2 (0.3%)	1 (0.3%)	1 (0.2%)	0 (0.0%)	0 (0.0%)	4 (0.2%)
Complete primary education	4 (0.6%)	2 (0.5%)	2 (0.5%)	2 (1.1%)	1 (1.0%)	11 (0.6%)
Incomplete high school	7 (1.1%)	5 (1.3%)	6 (1.4%)	2 (1.1%)	2 (1.9%)	22 (1.3%)
Complete high school	62 (10.0%)	40 (10.2%)	47 (11.1%)	20 (10.7%)	5 (4.9%)	174 (10.1%)
Higher education (or studying)	277 (44.7%)	148 (37.9%)	180 (42.4%)	75 (40.1%)	51 (49.5%)	731 (42.4%)
Postgraduate studies	268 (43.2%)	195 (49.9%)	189 (44.5%)	88 (47.1%)	44 (42.7%)	784 (45.4%)
Have you experienced symptoms of COVID-19?						
No	524 (84.5%)	309 (79.0%)	378 (88.9%)	166 (88.8%)	87 (84.5%)	1464 (84.8%)
Yes	96 (15.5%)	82 (21%)	47 (11.1%)	21 (11.2%)	16 (15.5%)	262 (15.2%)
Did you have a confirmed diagnosis for COVID-19?						
No	609 (98.2%)	378 (96.7%)	418 (98.4%)	182 (97.3%)	103 (100.0%)	1690 (97.9%)
Yes	11 (1.8%)	13 (3.3%)	7 (1.6%)	5 (2.7%)	0 (0%)	36 (2.1%)
Are you a health professional?						
No	399 (64.4%)	241 (61.6%)	263 (61.9%)	118 (63.1%)	65 (63.1%)	1086 (62.9%)
Yes	221 (35.6%)	150 (38.4%)	162 (38.1%)	69 (36.9%)	38 (36.9%)	640 (37.1%)
If so, are you assisting people with COVID-19?						
No	150 (67.9%)	100 (66.7%)	120 (74.1%)	58 (84.1%)	27 (71.1%)	455 (71.1%)
Yes	71 (32.1%)	50 (33.3%)	42 (25.9%)	11 (15.9%)	11 (28.9%)	185 (28.9%)

**Table 2:** Comparison of sociodemographic variables of interest by the level of physical activity.

Variables	Physical Activity Levels					Total	*p*-Value *
	1	2	3	4	5		
Education level according to the Brazilian standard							
Incomplete elementary school	2 (50.0%)	1 (25.0%)	1 (25.0%)	0 (0.0%)	0 (0.0%)	4 (100.0%)	0.8428
Complete primary education	4 (36.4%)	2 (18.2%)	2 (18.2%)	2 (18.2%)	1 (9.1%)	11 (100.0%)	
Incomplete high school	7 (31.8%)	5 (22.7%)	6 (27.3%)	2 (9.1%)	2 (9.1%)	22 (100.0%)	
Complete high school	62 (35.6%)	40 (23.0%)	47 (27.0%)	20 (11.5%)	5 (2.9%)	174 (100.0%)	
Higher education (or studying)	277 (37.9%)	148 (20.2%)	180 (24.6%)	75 (10.3%)	51 (7.0%)	731 (100.0%)	
Postgraduate studies	268 (34.2%)	195 (24.9%)	189 (24.1%)	88 (11.2%)	44 (5.6%)	784 (100.0%)	
Total	620 (35.9%)	391 (22.7%)	425 (24.6%)	187 (10.8%)	103 (6.0%)	1726 (100.0%)	
Have you experienced symptoms of COVID-19?							
No	524 (35.8%)	309 (21.1%)	378 (25.8%)	166 (11.3%)	87 (5.9%)	1464 (100.0%)	0.0012
Yes	96 (36.6%)	82 (31.3%)	47 (17.9%)	21 (8.0%)	16 (6.1%)	262 (100.0%)	
Total	620 (35.9%)	391 (22.7%)	425 (24.6%)	187 (10.8%)	103 (6.0%)	1726 (100.0%)	
Did you have a confirmed diagnosis for COVID-19?							
No	609 (36.0%)	378 (22.4%)	418 (24.7%)	182 (10.8%)	103 (6.1%)	1690 (100.0%)	0.1886
Yes	11 (30.6%)	13 (36.1%)	7 (19.4%)	5 (13.9%)	0 (0.0%)	36 (100.0%)	
Total	620 (35.9%)	391 (22.7%)	425 (24.6%)	187 (10.8%)	103 (6%)	1726 (100.0%)	
Are you a health professional?							
No	399 (36.7%)	241 (22.2%)	263 (24.2%)	118 (10.9%)	65 (6.0%)	1086 (100.0%)	0.9062
Yes	221 (34.5%)	150 (23.4%)	162 (25.3%)	69 (10.8%)	38 (5.9%)	640 (100.0%)	
Total	620 (35.9%)	391 (22.7%)	425 (24.6%)	187 (10.8%)	103 (6.0%)	1726 (100.0%)	
If so, are you working with people with COVID-19?							
No	150 (33.0%)	100 (22.0%)	120 (26.4%)	58 (12.7%)	27 (5.9%)	455 (100.0%)	0.0639
Yes	71 (38.4%)	50 (27.0%)	42 (22.7%)	11 (5.9%)	11 (5.9%)	185 (100.0%)	
Total	221 (34.5%)	150 (23.4%)	162 (25.3%)	69 (10.8%)	38 (5.9%)	640 (100.0%)	

* Likelihood Ratio Test.
